# Cumulative Low-Dose-Rate Radiation Induces Oxidative Stress, Apoptosis, and Fibrosis in Mouse Testis

**DOI:** 10.3390/antiox14081028

**Published:** 2025-08-21

**Authors:** Eun-Jin Kim, Anjas Happy Prayoga, Jina Ha, Deok Gyeong Kang, Jinsung Yang, Sohi Kang, Jin-Mok Kim, Byeonggyu Ahn, Dang Long Cao, Seung Pil Yun, Bo Hyun Lee, Joong-Sun Kim, Dawon Kang

**Affiliations:** 1 Department of Physiology, College of Medicine, Gyeongsang National University, Jinju 52727, Republic of Korea; eunjin1981@hanmail.net (E.-J.K.); anjasprayoga712@gmail.com (A.H.P.); hjn47272@naver.com (J.H.); dksqudrb1184@naver.com (B.A.); caodanglong97@gmail.com (D.L.C.); blee@gnu.ac.kr (B.H.L.); 2 Institute of Medical Sciences, Gyeongsang National University, Jinju 52727, Republic of Korea; jyang@gnu.ac.kr (J.Y.); anakang@gnu.ac.kr (S.K.); spyun@gnu.ac.kr (S.P.Y.); 3 Department of Convergence Medical Science, Gyeongsang National University, Jinju 52727, Republic of Korea; 4 Department of Biochemistry, College of Medicine, Gyeongsang National University, Jinju 52727, Republic of Korea; duckkang@naver.com; 5 Department of Anatomy, College of Medicine, Gyeongsang National University, Jinju 52727, Republic of Korea; 6 Department of Clinical Laboratory Science, Masan University, Changwon 2640, Republic of Korea; jmkim@masan.ac.kr; 7 Department of Pharmacology, College of Medicine, Gyeongsang National University, Jinju 52727, Republic of Korea; 8 College of Veterinary Medicine, Chonnam National University, Gwangju 61186, Republic of Korea

**Keywords:** epididymis, fibrosis, low-dose-rate radiation, reactive oxygen species, testis

## Abstract

Ionizing radiation is a well-known environmental stressor capable of generating excessive reactive oxygen species (ROS), leading to oxidative damage in sensitive tissues, including the reproductive system. While oxidative stress is increasingly implicated in male reproductive dysfunction, the long-term effects of low-dose-rate (LDR) radiation on testicular structure and oxidative status remain underexplored. In this study, mice were exposed to continuous LDR radiation (0.39, 1.29, and 3.46 mGy/h) for 21 days to assess testicular histopathology and oxidative status. Although testis weight did not significantly differ among groups, histological analysis revealed basal membrane disruption and reduced spermatogenic cell populations in irradiated groups. Masson’s Trichrome and Sirius Red staining demonstrated dose-dependent collagen deposition, indicating progressive testicular fibrosis. TUNEL assays confirmed increased germ cell apoptosis in the mid- and high-dose-rate groups. ROS levels were significantly elevated only in the highest-dose group, suggesting a threshold-dependent oxidative stress response. These findings indicate that chronic LDR radiation induces testicular damage primarily through apoptosis and fibrosis, with oxidative stress potentially contributing at higher exposure levels.

## 1. Introduction

Radiation exposure, whether from environmental, workplace, or medical sources, has significant health implications, with its effects increasing in proportion to dose and dose rate [1,2]. Ionizing radiation disrupts normal cellular functions, including metabolism, proliferation, and differentiation, leading to mutations, apoptosis, necroptosis, and senescence in radiation-sensitive cells [3]. While high-dose radiation is known to induce severe tissue damage, the effects of low-dose radiation (LDR, ≤0.2 Gy) remain controversial [4]. Some studies suggest potential beneficial effects, yet the cumulative effect of prolonged low-dose exposure remains inadequately understood [5]. Even with the same cumulative dose, biological and histological responses vary depending on exposure duration and rate, influencing damage and defense mechanisms [4,5,6,7].

The male reproductive system is particularly vulnerable to ionizing radiation due to the high sensitivity of testicular tissue [4,8]. Even low doses can disrupt spermatogenesis, with as little as 0.1 Gy impairing sperm production and potentially leading to temporary or permanent infertility [9]. Structural abnormalities, particularly within the seminiferous tubules, further highlight the testis’s susceptibility to radiation-induced damage. While short-term low-dose-rate exposure has minimal effects on testicular histology [4,10], prolonged exposure may amplify oxidative stress, fibrosis, and apoptosis [11,12]. Continuous LDR radiation exposure (2 Gy for 21 days at 3.49 mGy/h) has been shown to reduce testicular weight, disrupt seminiferous tubule integrity, and increase oxidative stress and epigenetic markers [4], all of which may contribute to fibrotic progression.

Fibrosis involves the replacement of normal tissue with disorganized collagen fibers and excessive extracellular matrix (ECM), leading to tissue remodeling, organ dysfunction, and increased morbidity. Radiation-induced fibrosis causes irreversible damage [11,12], yet testicular fibrosis from radiation exposure remains poorly studied. In fibrotic testes, blood–testis barrier disruption depletes spermatogenic cells, reduces spermatogenesis, and increases peritubular myoid cells and fibroblasts. Interstitial immune cell infiltration and vascular inflammation further contribute to ECM accumulation, exacerbating fibrosis and ultimately leading to irreversible male infertility [13]. Although direct research on LDR radiation-induced testicular fibrosis is limited, studies on testicular structure and function suggest that prolonged radiation exposure induces oxidative stress and histological damage, possibly contributing to fibrotic changes [4,5,6,7].

This study investigated the effects of LDR radiation exposures at 3.46, 1.29, and 0.39 mGy/h for 21 days on the testes of C57BL/6 mice, focusing on testicular fibrosis. By comparing dose-dependent changes, our findings provide critical insights into the cumulative risks of long-term LDR radiation exposure and its potential impact on reproductive health.

## 2. Materials and Methods

### 2.1. Animals

Eight-week-old male C57BL/6 mice were obtained from the Central Lab Animal Inc. (Seoul, Republic of Korea) and housed in a specific pathogen-free facility. Environmental conditions were maintained at 23 ± 2 °C with 50 ± 5% relative humidity, artificial lighting from 08:00 to 20:00, and 13–18 air changes per hour. Mice were provided with a standard laboratory diet. All experimental procedures followed the National Institutes of Health Guide for the Care and Use of Laboratory Animals (NIH Publications No. 8023, 8th edition, revised 2011) [14] and were approved by the Institutional Animal Care and Use Committee (IACUC) of the Dongnam Institute of Radiological and Medical Sciences (DIRAMS) and Chonnam National University (approval no. CNU IACUC-YB-2024-172).

### 2.2. Radiation Exposure

**Figure 1:**
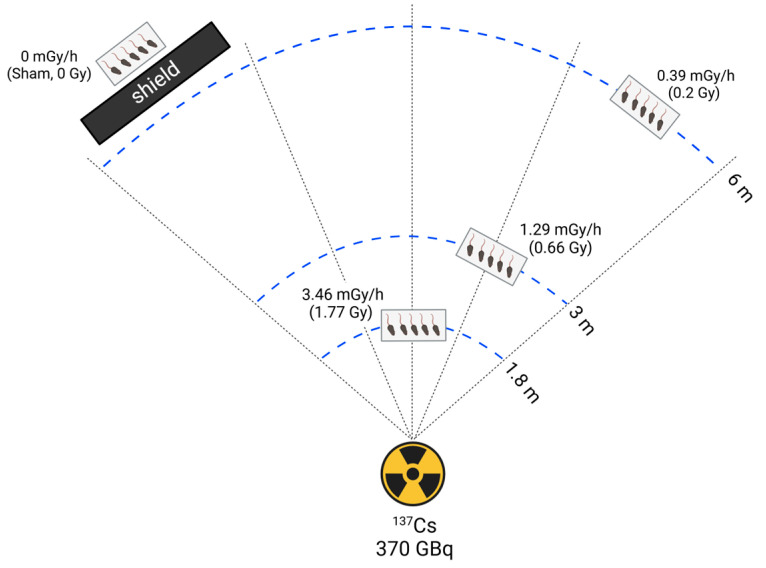
Schematic representation of the experimental setup for long-term low-dose-rate radiation exposure in mice.

Mice were randomly assigned to four groups (n = 5 per group): sham, 0.39, 1.29, and 3.46 mGy/h radiation exposure. Low-dose-rate (LDR) irradiation was performed following a previously described method [14] using a ^137^Cs source (370 GBq) at the DIRAMS LDR irradiation facility. Mice were continuously exposed for 21 days, except during routine cage cleaning and feeding. Radiation doses were assigned based on cage distance from the source. The sham group, which served as the negative control, was handled identically but without radiation exposure. The 0.39, 1.29, and 3.46 mGy/h groups were positioned at 6 m, 3 m, and 1.8 m from the radiation source, respectively. Over a 21-day exposure period, the total absorbed doses were 0.2 Gy, 0.66 Gy, and 3.46 mGy/h for the 0.39, 1.29, and 3.46 mGy/h groups, respectively. A shielding barrier was employed to completely block radiation exposure in the sham group (Figure 1). After irradiation, mice were sacrificed, and tissue samples were collected for analysis.

### 2.3. Chemicals

All chemicals used in this study were tissue culture-grade and, unless otherwise specified, were obtained from Sigma Chemical Co. (St. Louis, MO, USA).

### 2.4. Testis and Epididymis Isolation and Histological Staining

Testes and epididymes were collected, fixed in 4% paraformaldehyde at 4 °C overnight, rinsed in 0.1 M PBS, and embedded in paraffin. Paraffin-embedded testicular tissues were sectioned into 5 μm thick slices, mounted on gelatin-coated slides, and air-dried. The sections were deparaffinized in xylene (twice for 5 min each), rehydrated through a graded ethanol series (100%, 95%, 80%, 70%), and rinsed in double-distilled water.

#### 2.4.1. Hematoxylin and Eosin (H&E) Staining

The H&E staining was carried out as described previously [15]. The rehydrated tissue slides were stained with hematoxylin for 5 min and eosin for 1 min. Sections were then dehydrated in graded ethanol (70–100%), cleared in xylene, and mounted with Permount (Fisher Chemical, Geel, Belgium). Images were captured using a BX61VS microscope (Olympus, Tokyo, Japan), and five sections per sample were analyzed.

#### 2.4.2. Sirius Red Staining

The rehydrated tissue slides were incubated with Picro-Sirius Red solution (Abcam, Cambridge, UK) for 60 min at room temperature. After rinsing in 0.5% acetic acid and absolute alcohol, sections were dehydrated and mounted with synthetic resin. Sirius Red-positive areas were quantified using Fiji (ImageJ 2.9.0; NIH, Bethesda, MD, USA).

#### 2.4.3. Masson’s Trichrome Staining

The rehydrated tissue slides were mordanted in Bouin’s solution (56–64 °C) for 60 min. After cooling and rinsing, sections were stained with hematoxylin for 5 min, followed by Biebrich Scarlet-Acid Fuchsin for 15 min. Collagen fibers were differentiated using phosphotungstic/phosphomolybdic acid, then stained with Aniline Blue for 5–10 min. Sections were treated with 1% acetic acid for contrast enhancement, dehydrated in ethanol, cleared in xylene, and mounted with synthetic resin.

### 2.5. TUNEL Staining

Apoptotic signals in the testes were detected using the In Situ Cell Death Detection Kit, POD (Roche, Mannheim, Germany), following the manufacturer’s protocol. Tissue sections were permeabilized with Proteinase K (10–20 μg/mL) at 37 °C for 20 min and incubated in 3% H_2_O_2_/methanol to block endogenous peroxidase. After PBS washes, 50 µL of TUNEL reaction mixture (Enzyme + Label Solution) was applied and incubated at 37 °C for 1 h in the dark. Negative controls received Label Solution only; positive controls were pretreated with DNase I (3 U/mL) for 10 min. Converter-POD was added and incubated for 1 h at 37 °C. Signals were visualized with DAB and counterstained with DAPI. Slides were dehydrated, mounted, and imaged under a light microscope. Green fluorescence before POD development was detected using a confocal laser scanning microscope (Zeiss LSM-900, Carl Zeiss AG, Oberkochen, Germany).

### 2.6. Comet Assay

Microscope slides were precoated with 1% normal-melting-point (NMP) agarose and air-dried overnight. Frozen testis tissue was thawed in ice-cold PBS, minced, and filtered through a 70 μm cell strainer to obtain a single-cell suspension. Cells were centrifuged at 300× *g* for 5 min at 4 °C, and the pellet was resuspended in 500 μL PBS. An equal volume of 1% low-melting-point (LMP) agarose (final 0.5%) at 37 °C was added and gently mixed. The suspension was pipetted onto agarose-coated slides, covered with coverslips, and solidified at 4 °C for 5 min. After removing coverslips, slides were incubated in lysis buffer (2.5 M NaCl, 100 mM EDTA, 10 mM Tris-HCl, 1% Triton X-100, 10% DMSO) for 1 h at 4 °C. Slides were rinsed, then placed in alkaline buffer (300 mM NaOH, 1 mM EDTA, pH 13) for 40 min at 4 °C to unwind DNA, followed by electrophoresis at 25 V and 300 mA for 30 min. Afterward, slides were neutralized in TBE buffer (pH ~ 8) for 10 min three times. DNA was stained with 600 μL GreenStar™ Nucleic Acid Staining Solution (Bioneer, Daejeon, Republic of Korea) for 5 min in the dark, rinsed, and visualized under a fluorescence microscope. Tail DNA (%) was quantified using Comet Score 2.0.

### 2.7. Measurement of Total Free Radical Activity

Per the manufacturer’s instructions, total free radical activity was measured using the Oxiselect™ In Vitro ROS/RNS Assay Kit (Cell Biolabs, San Diego, CA, USA). DCF standards (0–10 μM) and tissue lysates (50 µL) were loaded into a 96-well black-bottom fluorescence plate (Nunclon™, Thermo Fisher Scientific, Roskilde, Denmark), followed by the addition of 50 µL of 1× catalyst. After a 5 min incubation at room temperature, 100 µL of DCFH solution was added. The plate was incubated in the dark for 45 min at room temperature. Fluorescence was read at 480 nm excitation and 530 nm emission using a GloMax^®^ Explorer (Promega, Madison, WI, USA). This assay detects dichlorodihydrofluorescein DiOxyQ (DCFH-DiOxyQ) oxidation to fluorescent DCF in the presence of ROS/RNS, with signal intensity proportional to total oxidant levels.

### 2.8. Isolation of Total RNA and Reverse Transcriptase–Polymerase Chain Reaction (RT-PCR)

Total RNA was extracted from testes using TRIzol™ Reagent (Invitrogen, Carlsbad, CA, USA), and cDNA was synthesized with 3 μg of total RNA using the DiaStart™ RT Kit (SolGent, Daejeon, Republic of Korea). PCR amplification was conducted using first-strand cDNA, *Taq* polymerase (G-Taq, Cosmo Genetech, Seoul, Republic of Korea), and specific primers for mouse *ACTA2* (*BC064800.1*, forward: 5′-TCATTGGGATGGAGTCAGCG-3′, reverse: 5′-AATGCCTGGGTACATGGTGG-3′). GAPDH (GU214026.1, forward: 5′-ACCCAGAAGACTGTGGATGG-3′, reverse: 5′-CACATTGGGGGTAGGAACAC-3′) was used as a loading control. Real-time PCR was conducted using the TOPreal^TM^ SYBR Green 2x PreMix (Enzynomics, Daejeon, Republic of Korea) on a LightCycler^®^ 480 II/96 (Roche Diagnostics Ltd., Rotkreuz, Switzerland). The protocol included an initial denaturation at 95 °C for 5 min, followed by 45 cycles of denaturation at 95 °C for 30 s, annealing at 60 °C for 30 s, and extension at 72 °C for 30 s. Real-time PCR data were analyzed statistically with the 2^−ΔΔCt^ method to determine mRNA level changes, with mRNA expression of target genes normalized to GAPDH levels.

### 2.9. Statistical Analysis

The data are represented as the mean ± S.D. Significant differences between groups were evaluated using a one-way ANOVA/Bonferroni test (OriginPro2020, OriginLab Corp., Northampton, MA, USA). A value of *p* < 0.05 was considered to be significant.

## 3. Results

### 3.1. Effect of Low-Dose-Rate (LDR) Radiation on Testicular Oxidative Stress

**Figure 2:**
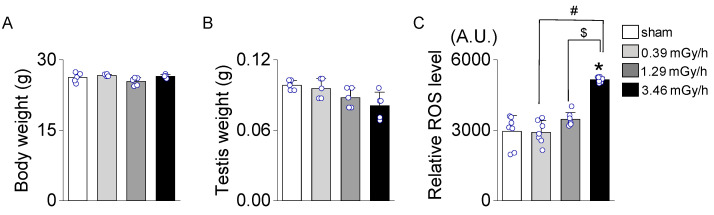
Relative reactive oxygen species (ROS) levels in sham and low-dose-rate (LDR) radiation-exposed groups (0.39, 1.29, and 3.46 mGy/h). (**A**) Body weight. (**B**) Testis weight. (**C**) ROS levels were measured and expressed in arbitrary units (A.U.). Data are presented as mean ± standard deviation (S.D.). Each circle represents an individual sample in panels (**A**,**B**), and an individual data point in panel (**C**). * *p* < 0.05 vs. sham; ^#^ *p* < 0.05 vs. 0.39 mGy/h; ^$^ *p* < 0.05 vs. 1.29 mGy/h.

No significant differences in body weight were observed among the experimental groups (sham, 0.39, 1.29, and 3.46 mGy/h; Figure 2A). Similarly, testis weight did not differ significantly across the groups (Figure 2B). However, the relative levels of reactive oxygen species (ROS) were significantly elevated in the 3.46 mGy/h group compared to the sham group (*p* < 0.05, Figure 2C).

### 3.2. Histopathological Analysis of Testicular Tissue

**Figure 3:**
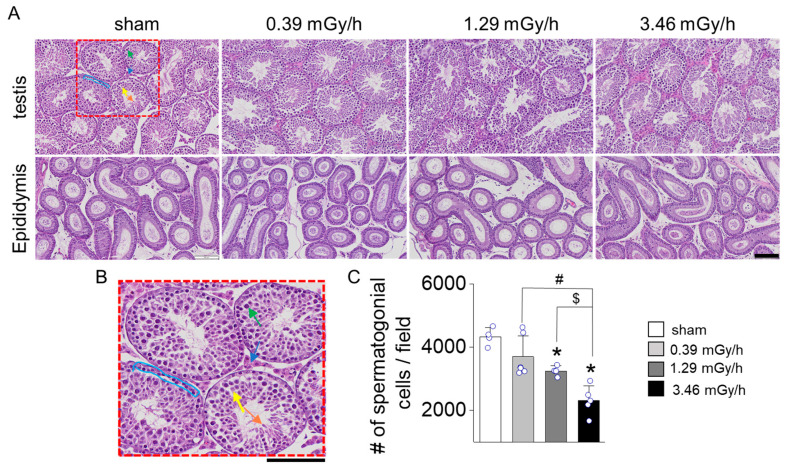
Histological analysis of seminiferous tubules and epididymis in mouse testes exposed to low-dose-rate radiation. (**A**) Representative H&E-stained sections from the sham group (0 Gy) and radiation-exposed groups (0.39, 1.29, and 3.46 mGy/h) are shown. The upper panel displays the overall structure of seminiferous tubules, while the lower panel depicts the head of the epididymis. (**B**) The image displays key structural features within the seminiferous epithelium expanded from the red-dotted box in panel (**A**). The green arrow indicates spermatocytes, the yellow arrow points to Sertoli cells along the basal membrane, and the orange arrow marks elongating spermatids. The light blue bracket outlines the spermatogonia region, while the blue arrow denotes Leydig cells in the interstitial space. Scale bars: 100 μm. (**C**) Quantification of spermatogonia cell numbers per image field. Data are presented as mean ± standard deviation (S.D.). Each circle represents an individual sample. * *p* < 0.05 vs. sham; ^#^ *p* < 0.05 vs. 0.39 mGy/h; ^$^ *p* < 0.05 vs. 1.29 mGy/h.

Hematoxylin and eosin (H&E) staining revealed dose-dependent structural damage to the seminiferous tubules and a reduction in spermatogenic cells in all radiation-exposed groups. In the sham group, the seminiferous tubules appeared intact and well-preserved, with a continuous basal membrane and no observable disruptions. Spermatogenic cells were abundant and arranged in distinct layers. In contrast, the radiation-exposed groups exhibited moderate structural damage, including thinning and disruption of the basal membrane (Figure 3A). In addition, although it is unclear whether there is a significant difference in the diameter of the seminiferous tubules in the testis, exposure to LDR radiation appears to reduce the epithelial cell density in a dose-dependent manner. No notable structural differences were observed in the epididymis among the groups. Representative structures within the seminiferous tubules are shown in the magnified image, including spermatocytes, Sertoli cells, spermatids, spermatogonia, and Leydig cells in the interstitial space (Figure 3B). The number of spermatogenic cells was noticeably reduced in the 3.46 mGy/h group compared to the sham group (*p* < 0.05, Figure 3C). The 3.46 mGy/h group also showed visible disorganization of cell layers in the epididymis.

### 3.3. Fibrosis Assessment Following LDR Radiation Exposure

**Figure 4:**
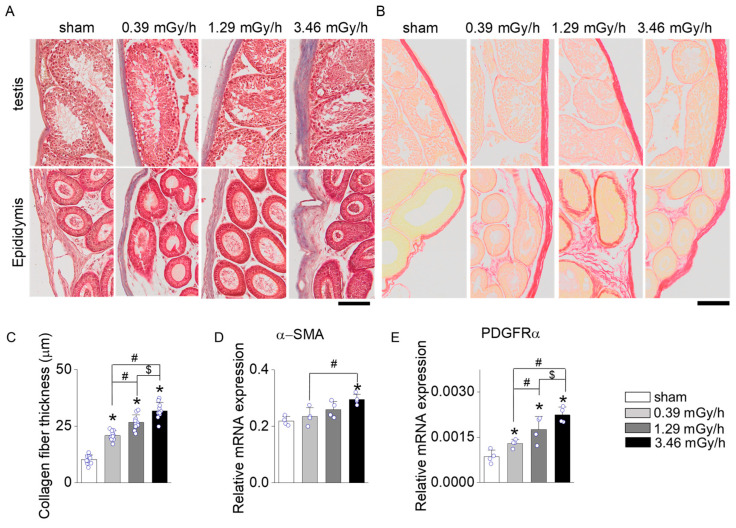
Fibrosis analysis in testis and epididymis following LDR radiation exposure. (**A**) Masson’s Trichrome (M&T) and (**B**) Sirius Red staining of the testis and epididymis from the sham and LDR-exposed groups (0.39, 1.29, and 3.46 mGy/h). (**C**) Quantification of collagen accumulation based on histological staining. (**D**) mRNA expression levels of α-SMA and (**E**) PDGFR-α. Data are presented as mean ± standard deviation (S.D.). Each circle represents an individual sample. Scale bar: 100 μm. * *p* < 0.05 vs. sham; ^#^ *p* < 0.05 vs. 0.39 mGy/h; ^$^ *p* < 0.05 vs. 1.29 mGy/h.

Masson’s Trichrome and Sirius Red staining were performed on testis and epididymis tissues following LDR radiation exposure to assess radiation-induced fibrosis. In the sham group, collagen deposition was minimal, confined to the basal membrane and interstitial regions of the seminiferous tubules in the testis and the connective tissue surrounding the epididymal ducts (Figure 4A,B). In contrast, mice exposed to 0.39, 1.29, and 3.46 mGy/h showed a dose-dependent increase in collagen accumulation (Figure 4A–C). The 3.46 mGy/h group exhibited the most pronounced collagen deposition, with visibly thickened collagen fibers in both the testis and epididymis. Furthermore, expression analyses of α-smooth muscle actin (α-SMA, *ACTA2*) and platelet-derived growth factor receptor alpha (*PDGFRα*) in testicular tissue revealed significant upregulation of these fibrosis-associated genes in the 3.46 mGy/h group compared to the sham group (Figure 4D,E).

### 3.4. Increase in Apoptotic Signal in LDR-Exposed Experimental Groups

**Figure 5:**
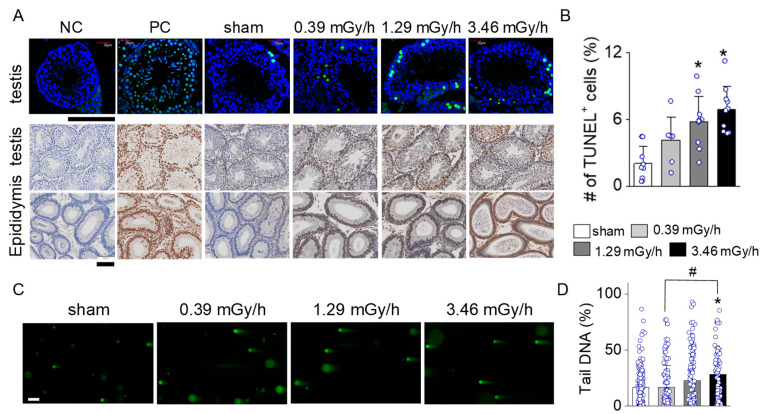
TUNEL staining of testicular and epididymal tissues showing radiation-induced apoptosis at varying doses. (**A**) Representative images of testicular (**upper** panels) and epididymal (**lower** panels) sections are shown for the negative control (NC), positive control (PC), sham, 0.2 Gy, 0.66 Gy, and 3.46 mGy/h groups. TUNEL-positive cells (green fluorescence, DAB brown signal) indicate apoptotic nuclei in the testis. (**B**) Quantification of the percentage of TUNEL-positive cells in testicular tissues across groups. (**C**) Representative fluorescence images from the comet assay of testicular cells following irradiation at 0 (sham), 0.39, 1.29, and 3.46 mGy/h. (**D**) Quantification of DNA damage based on the percentage of tail DNA from comet images. Each circle represents individual data points. Scale bars: 100 µm. * *p* < 0.05 vs. sham; ^#^ *p* < 0.05 vs. 0.39 mGy/h.

TUNEL staining assessed apoptosis in testicular and epididymal tissues in the sham and LDR-exposed groups (0.39, 1.29, and 3.46 mGy/h). As shown in Figure 5A, the TUNEL-positive cells (green fluorescence in upper panels, brown signal in lower panels) in testicular tissue increased progressively with radiation dose. The sham group exhibited minimal TUNEL staining, similar to the negative control (NC). The 1.29 mGy/h and 3.46 mGy/h groups showed a marked increase in TUNEL-positive cells compared to the sham. The epididymal tissues followed a similar trend, with increased TUNEL staining intensity at higher radiation doses, particularly in the 3.46 mGy/h group. The bar graph shows a statistically significant increase in the percentage of TUNEL-positive cells in the testis of the 1.29 mGy/h and 3.46 mGy/h groups compared to the sham group (*p* < 0.05; Figure 5B).

The comet assay further confirmed the presence of DNA strand breaks, indicative of radiation-induced genotoxicity. Specifically, the 3.46 mGy/h group demonstrated a significant increase in DNA damage compared to the sham and 0.39 mGy/h groups (*p* < 0.05; Figure 5C,D). These findings suggest that exposure to 3.46 mGy/h LDR radiation induces both apoptotic and non-apoptotic forms of DNA damage in testicular tissue.

## 4. Discussion

This study demonstrates that chronic LDR radiation exposure induces testicular damage in a dose-dependent manner, with distinct threshold responses across different biological markers. While body and testis weights remained unchanged, histological and molecular assessments revealed significant alterations in testicular architecture and function, particularly at higher radiation dose rate.

Oxidative stress, assessed by ROS levels, was significantly elevated only in the 3.46 mGy/h group, indicating a threshold effect. This aligns with previous studies suggesting that prolonged or high-intensity radiation is required to overcome antioxidant defenses and trigger measurable oxidative damage [16,17]. While irradiation dose rates vary across studies, our study employed one of the lowest LDR conditions reported to date. Despite this lower dose rate, ROS levels were markedly elevated, indicating that even minimal LDR radiation can induce significant oxidative stress in testicular tissue. ROS plays a central role in radiation-induced tissue damage by promoting DNA strand breaks, lipid peroxidation, and cellular senescence, ultimately leading to fibrotic remodeling [12,18]. The absence of elevated ROS in the lower-dose-rate groups (0.39 and 1.29 mGy/h) implies that compensatory mechanisms may buffer oxidative stress at sub-threshold exposures. However, once the radiation dose exceeds a critical level, as seen in the 3.46 mGy/h group, these defense mechanisms may be overwhelmed, leading to excessive ROS accumulation and downstream pathological changes [19].

Apoptosis of spermatogenic cells, especially spermatogonia, was most prominent at 3.46 mGy/h, indicating that these highly proliferative germ cells are vulnerable to sustained oxidative and genotoxic stress. This aligns with previous studies showing a dose-dependent sensitivity of spermatogonia to radiation exposure [20,21]. TUNEL staining revealed increased apoptotic cells in the 1.29 and 3.46 mGy/h groups, while the comet assay detected significant DNA damage only at 3.46 mGy/h. These findings reflect the complementary nature of these assays; TUNEL detects apoptotic DNA fragmentation, while the comet assay is more sensitive to early-stage genotoxicity [22]. Detecting apoptosis at 1.29 mGy/h suggests moderate LDR exposure can activate cell death pathways without measurable oxidative stress.

Interestingly, collagen deposition was observed even at the lowest radiation dose-rate, suggesting that testicular fibrosis can be initiated independently of pronounced oxidative stress or apoptosis. As reported in other chronic LDR exposure models, these early fibrotic changes may reflect subclinical inflammation or low-grade tissue injury [7]. This indicates a two-phase response: collagen accumulation may represent an early and progressive reaction to radiation, while oxidative stress and apoptosis signify more advanced tissue damage [23]. In contrast, the upregulation of α-SMA, a marker of myofibroblast activation, was confined to the highest dose rate, reinforcing the association between higher radiation exposure, cell death, and active fibrotic remodeling [24,25].

Apoptosis promotes fibrosis by releasing pro-fibrotic mediators such as TGF-β and PDGF, which activate myofibroblasts and stimulate ECM production [26,27]. Moreover, insufficient clearance of apoptotic cells can lead to chronic inflammation, further amplifying fibrotic responses [28]. Standard testicular fibrosis models include experimental autoimmune orchitis, which causes chronic immune-mediated damage and severe interstitial fibrosis [13,29], and the CCl_4_-induced model, which causes oxidative injury through metabolic activation and ROS generation [30,31,32]. These models feature strong inflammatory responses and upregulation of TNF-α, TGF-β, α-SMA, and collagen I.

In contrast, our LDR radiation model induces fibrosis through sustained oxidative stress without immune sensitization or hepatotoxicity. Over 21 days of continuous exposure, we observed spermatogonial apoptosis, ECM remodeling, and upregulation of fibrotic markers, including α-SMA and PDGFRα. Collagen accumulation was predominantly interstitial, with relatively preserved seminiferous architecture and minimal immune cell infiltration, suggesting milder structural disruption than in classical models [13,29,30,31,32]. Though radiation is often considered an acute insult, the 21-day exposure duration in our study aligns with subchronic fibrosis protocols such as those using CCl_4_ (typically 21–28 days) [30,31,32]. Thus, the fibrotic changes observed here reflect a progressive, subchronic response. Chronic LDR exposure promotes persistent ROS production, low-grade inflammation, and fibroblast-to-myofibroblast transition. This leads to ECM accumulation—mechanisms that parallel those in chemical fibrosis models but with slower onset and reduced cytotoxicity [17]. While the study focused on testicular endpoints, our findings establish foundational evidence linking LDR radiation to apoptosis-driven fibrotic remodeling. Future work should expand on these results by investigating upstream signaling pathways, immune responses, and comparative analyses with established fibrosis models.

We additionally analyzed bronchoalveolar lavage fluid (BALF), peritoneal lavage fluid (PLF), and blood to evaluate systemic toxicity. No inflammatory responses were observed in BALF or blood, and liver enzymes (ALT, AST) remained within normal ranges, suggesting that testicular fibrosis is a localized response rather than a manifestation of systemic damage. These findings are consistent with previous studies reporting organ-specific sensitivity to radiation exposure [33,34].

Our findings reveal a dose-dependent pattern of testicular responses to LDR radiation. Collagen deposition was observed across all exposure levels, suggesting it may be an early and general response to radiation. In contrast, oxidative stress, apoptosis, and myofibroblast activation became prominent only at higher dose rates, indicating a threshold-dependent progression of tissue damage. These results raise concerns about the cumulative effects of chronic LDR exposure, which may gradually induce structural remodeling and impair testicular function, even at doses relevant to environmental or occupational settings. The strong association between elevated ROS and upregulation of fibrotic markers such as α-SMA and PDGFRα further highlights oxidative stress as a key driver of fibrosis under high-dose-rate conditions.

## 5. Conclusions

This study demonstrates that prolonged exposure to LDR radiation, at levels comparable to environmental or occupational settings, can dose-dependently induce testicular fibrosis and cellular damage. While collagen deposition occurred even at the lowest exposure level (0.39 mGy/h), more pronounced structural damage and germ cell apoptosis were evident at higher doses. Oxidative stress, as indicated by significantly elevated ROS levels in the highest-dose-rate group (3.46 mGy/h), may contribute to the progression of fibrosis and cell death at higher exposure thresholds. These findings suggest chronic LDR radiation can disrupt testicular homeostasis through apoptosis and extracellular matrix remodeling, with oxidative stress potentially amplifying these effects under higher cumulative exposure. Our findings highlight the importance of further investigating the mechanisms of reproductive toxicity in populations exposed to long-term low-dose radiation.

## Data Availability

The data supporting this study’s findings are available on reasonable request from the corresponding author (D.K.). The article includes all relevant data supporting this study’s conclusions.

## Abbreviations

The following abbreviations are used in this manuscript:

LDR	Low Dose Rate
PDGFR	Platelet-Derived Growth Factor Receptor
ROS	Reactive Oxygen Species
SMA	Smooth Muscle Actin
